# Effect of digital hypothermia on lamellar inflammatory signaling in the euglycemic hyperinsulinemic clamp laminitis model

**DOI:** 10.1111/jvim.15835

**Published:** 2020-06-25

**Authors:** Simon M. Stokes, Teresa A. Burns, Mauria R. Watts, François‐René Bertin, Darko Stefanovski, Carlos E. Medina‐Torres, James K. Belknap, Andrew W. van Eps

**Affiliations:** ^1^ Australian Equine Laminitis Research Unit, School of Veterinary Science The University of Queensland Gatton Queensland Australia; ^2^ Department of Veterinary Clinical Sciences The Ohio State University College of Veterinary Medicine Columbus Ohio USA; ^3^ New Bolton Center, Department of Clinical Studies, School of Veterinary Medicine University of Pennsylvania Kennett Square Pennsylvania USA

**Keywords:** endocrinopathic, horse, hypothermia, inflammation, insulin

## Abstract

**Background:**

Continuous digital hypothermia (CDH) prevents lamellar failure in the euglycemic hyperinsulinemic clamp (EHC) model of laminitis, but the protective mechanisms are unclear.

**Hypothesis/Objectives:**

To determine if CDH inhibits lamellar inflammatory signaling in the EHC model of laminitis.

**Animals:**

Eight Standardbred horses.

**Methods:**

Prospective experimental study. Horses underwent an EHC, with 1 forelimb treated with CDH and the other kept at ambient temperature (AMB). Horses were euthanized 48 hours after initiation of the EHC and lamellar tissue was analyzed via polymerase chain reaction (pro‐inflammatory cytokine and chemokine genes—CXCL1, CXCL6, CXCL8, IL‐6, MCP‐1, MCP‐2, IL‐1β, IL‐11, cyclooxygenase 1 and 2, tumour necrosis factor‐alpha [TNF‐α], E‐selectin, and intercellular adhesion molecule‐1 [ICAM‐1]) and immunoblotting (phosphorylated and total signal transducer and activator of transcription 1 [STAT1] and STAT3).

**Results:**

Compared to AMB, lamellar messenger ribonucleic acid (mRNA) concentrations of CXCL6 (*P* =.02), CXCL8 (*P* = .008), IL‐6 (*P* = .008), IL‐1β (*P* = .008), IL‐11 (*P* = .008), and cyclooxygenase‐2 (*P* = .008) were decreased in CDH. Cyclooxygenase‐1 (*P* = .008) was increased in CDH, while CXCL1 (*P* = .15), MCP‐1 (*P* = .05), MCP‐2 (*P* = .46), TNF‐α (*P* = .05), E‐selectin (*P* = .15), and ICAM‐1 (*P* = .15) mRNA were not significantly different. Compared to AMB, lamellar concentration of total STAT3 protein was decreased in CDH (*P <* .001), but there was no change in phosphorylated STAT3 (P‐STAT3 [S727] *P* = .19; P‐STAT3 [Y705] *P* = .05). There was no change in lamellar concentrations of total STAT1 (*P* = .75) or phosphorylated STAT1 (P‐STAT1 [S727], *P* = .25; P‐STAT1 [Y701], *P* = .64).

**Conclusions and Clinical Importance:**

These data add further support for the use of CDH as a first aid treatment for severe acute laminitis associated with hyperinsulinemia in horses.

**Abbreviations**
AMB: ambient temperature
CDH: continuous digital hypothermia
cDNA: complementary deoxyribonucleic acid
COX‐1: cyclooxygenase‐1
COX‐2: cyclooxygenase‐2
CXCL1: C‐X‐C motif chemokine ligand‐1
CXCL6: C‐X‐C motif chemokine ligand‐6
CXCL8: C‐X‐C motif chemokine ligand‐8
EHC: euglycemic‐hyperinsulinemic clamp
ICAM‐1: intercellular adhesion molecule‐1
IGF‐1R: insulin‐like growth factor 1 receptor
IL‐1β: interleukin‐1β
IL‐6: interleukin‐6
IL‐11: interleukin‐11
MCP‐1: monocyte chemoattractant protein‐1
MCP‐2: monocyte chemoattractant protein‐2
mRNA: messenger ribonucleic acid
mTORC1: mammalian target of rapamycin complex‐1
PCR: polymerase chain reaction
RPS6: ribosomal protein S6
STAT1: signal transducer and activator of transcription‐1
STAT3: signal transducer and activator of transcription‐3
TBST: tris‐buffered saline plus Tween‐20
TNF‐α: tumor necrosis factor‐alpha

## 1 INTRODUCTION

Insulin dysregulation is a common contributor to most clinical cases of laminitis.^1^, ^2^ In addition, laminitis can be induced experimentally with hyperinsulinemia, as is demonstrated in horses undergoing a prolonged euglycemic hyperinsulinemic clamp (EHC).^3^, ^4^ Although this finding confirms that hyperinsulinemia is an important component of the pathogenesis, there is still uncertainty as to how hyperinsulinemia induces laminitis. Continuous digital hypothermia (CDH) has been shown to protect the lamellae during the EHC^3^ and therefore studies aimed at determining the nature of this protective effect could provide insight into the pathogenesis of insulin‐associated laminitis and facilitate the development of targeted therapeutics.

Recent research on the pathogenesis of endocrinopathic laminitis has focused on lamellar vascular derangements,^5^, ^6^ disturbances of glucose metabolism,^7^, ^8^ and lamellar events related to insulin‐like growth factor‐1 receptor (IGF‐1R) activation including cellular proliferation and loss of cell adhesion.^9^, ^10^ A role for IGF‐1R signaling has been supported by the presence of IGF‐1R on lamellar epithelial cells,^10^, ^11^ downregulation of lamellar IGF‐1R expression in horses undergoing an EHC,^12^ and also evidence of activated signaling pathways related to IGF‐1R (ie, the mammalian target of rapamycin complex‐1 [mTORC1]/ribosomal protein S6 [RPS6] signaling pathway).^9^ A recent study showed that insulin does not readily bind to lamellar IGF‐1R at relevant concentrations,^13^ questioning whether direct activation of IGF‐1R by insulin is responsible for activating lamellar signaling pathways related to IGF‐1R.^9^ Lamellar leukocyte infiltration^3^ and increased lamellar gene expression of pro‐inflammatory cytokines^14^ has been observed in the EHC model, suggesting a role for lamellar inflammation in the pathogenesis of insulin‐associated laminitis. Inflammation also provides an alternative, indirect explanation for activation of signaling pathways related to IGF‐1R in the EHC model, for example, via inflammatory mediators including interleukin‐6 (IL‐6) through the gp130 receptor.^15^

It is well established that CDH reduces lamellar injury in sepsis‐related laminitis and this protection has been partly attributed to an anti‐inflammatory effect.^16^, ^17^ In support of this, CDH has been shown to attenuate lamellar inflammation in sepsis‐associated laminitis models by reducing inflammatory cell infiltration^18^ and the expression of pro‐inflammatory cytokines, chemokines, cyclooxygenase‐2, and endothelial adhesion molecules.^16^ In addition, the effects of CDH on lamellar inflammatory signaling pathways has helped identify signaling pathways that could be exploited therapeutically.^15^, ^17^ It is currently unclear if CDH affects lamellar inflammation in the EHC model of endocrinopathic laminitis. We hypothesized that CDH would have a similar inhibitory effect on lamellar inflammation to that observed in sepsis‐associated laminitis models.^16^, ^17^ To investigate this, the messenger ribonucleic acid (mRNA) concentration of inflammatory mediators (pro‐inflammatory cytokines, chemokines, endothelial adhesion molecules, and cyclooxygenase enzymes) and protein concentration of inflammation‐associated proteins (STAT proteins) were quantified in the lamellar tissue of CDH‐treated and untreated feet from horses undergoing the EHC model of endocrinopathic laminitis.

## 2 MATERIALS AND METHODS

This project was approved by the University of Queensland Animal Ethics Committee that monitors compliance with the Animal Welfare Act (2001) and The Code of Practice for the care and use of animals for scientific purposes (current edition). All animals were monitored continuously by the investigators.

### 2.1 Animal protocol

Eight healthy Standardbred geldings (mean age: 6.3 ± 1.7 years; mean bodyweight: 447.8 ± 36.9 kg) recently retired (<4 weeks) from racing underwent laminitis induction using the EHC model. All horses were sound at the walk and had no gross or radiographic abnormalities of the feet. For the duration of the experiment, all horses were confined to stocks and received ad libitum access to alfalfa hay and water. An EHC was performed for 48 hours as previously described.^3^ Briefly, an intravenous bolus (45 mIU/kg bwt) of recombinant human insulin (Humulin‐R, Eli‐lily, West Ryde, Australia) diluted in 50 mL of 0.9% sodium chloride (Baxter, Old Toongabbie, Australia) was administered via a 14‐gauge jugular catheter and was immediately followed by a continuous intravenous infusion of insulin in 0.9% sodium chloride (Baxter) at a rate of 6 mIU/(kg bwt min). A continuous intravenous infusion of 50% glucose (Baxter) was administered concurrently, with the administration rate adjusted to maintain euglycemia (4.0 ± 1.0 mM/L). Blood glucose was measured using a portable glucometer (Roche Diagnostics, Indianapolis, Indiana) every 5 minutes until euglycemia was achieved for 30 minutes without the need to change the glucose infusion rate. Blood glucose was then measured every 30 minutes for the remainder of the experiment. For the duration of the experiment, horses were constantly monitored and vital signs were measured every 2 hours.

Thirty minutes before starting the EHC, 1 forelimb was randomly selected by coin toss to be cooled for the duration of the 48 hour EHC using a rubber boot (Bigfoot Ice boots, Esk, Australia) containing ice cubes and water. Ice was replenished as required to maintain a 50% ice, 50% water mixture to a level just distal to the carpus. The contralateral limb was kept at ambient temperature (AMB). Hoof wall surface temperature in CDH and AMB limbs was monitored using hoof wall thermistors attached to data‐logging devices as previously described.^16^

After 48 hours of the EHC, the horses were euthanized with pentobarbital sodium (20 mg/kg bwt IV). Dorsal lamellar tissue samples were rapidly dissected from the hoof and distal phalanx in each forelimb (AMB and CDH) from each study subject and were snap frozen in liquid nitrogen_._

### 2.2 RNA isolation and complementary DNA synthesis

Three separate sections of lamellar tissue from each forelimb of each horse were pulverized and total RNA was isolated using a guanidine thiocyanate spin column method (Agilent Technologies, West Cedar, Texas) with a DNase treatment to remove genomic DNA contamination. Messenger RNA was then isolated with a poly‐A tail streptavidin magnetic bead kit (Roche Diagnostics) and used to make complementary DNA (cDNA) using a total of 400 ng of mRNA. The cDNA was diluted at 1 : 5, 1 : 50, and 1 : 500 and frozen at −20°C until used for real‐time quantitative polymerase chain reaction (RT‐qPCR) analysis.

### 2.3 Real‐time polymerase chain reaction

Real‐time quantitative polymerase chain reaction was performed using a thermocycler (Roche LC 2.0; Roche Life Science, Indianapolis, Indiana) as previously described.^14^ Each set of reactions containing AMB and CDH lamellar tissue was run in duplicate and included double distilled water as a negative control, controls without added reverse transcriptase, and a standard curve made from serial dilutions (10^1^‐10^6^) of linearized vector containing the equine‐specific gene sequence of interest. Primers for the following genes were synthesized based on published equine genomic sequences using a commercial software program (DNASTAR, Madison, Wisconsin): C‐X‐C motif chemokine ligand 1 (CXCL1), CXCL6, interleukin 8 (IL‐8), IL‐6, monocyte chemoattractant protein 1 (MCP‐1), monocyte chemoattractant protein‐2 (MCP‐2), interleukin‐1β (IL‐1β), interleukin‐11 (IL‐11), tumour necrosis factor‐alpha (TNF‐α), cyclooxygenases 1 and 2 (COX‐1, COX‐2), intercellular adhesion molecule 1 (ICAM‐1), E‐selectin, and the reference genes β‐actin and β‐2 microglobulin. The qPCR data from the selected housekeeping genes (β‐actin and β‐2 microglobulin) were entered into a computer program (geNorm) to test the suitability of each gene as a housekeeping gene for the lamellar tissue samples. Both genes were determined to be satisfactory by the program and a geometric mean was obtained from the 2 genes' data in order to generate a normalization factor for each lamellar tissue sample. The amplification data for the genes of interest were then divided by the unique normalization factor of the selected housekeeping genes in each lamellar tissue sample, creating a normalized copy number value. The data are reported as either absolute copy number or fold‐change from AMB values (calculated by dividing the normalized CDH expression values by the normalized AMB values for each gene).

### 2.4 Protein extraction

Protein was extracted from snap‐frozen lamellar samples as previously described.^9^, ^14^ Briefly, all samples underwent an initial pulverization step before homogenization on ice in a commercially available lysis buffer (Thermo Scientific, Waltham, Massachusetts) with the addition of protease and phosphatase inhibitors and phenylmethylsulfonyl fluoride. Samples were then incubated on ice for 30 minutes. Supernatant was separated by centrifugation, and protein concentration was quantified using the Bradford method. Protein samples were stored at −80°C until analysis.

### 2.5 Immunoblotting

The concentration of phosphoprotein was assessed as previously described^14^ via Western immunoblot analysis of lamellar samples using commercially available antibodies raised against STAT1 P‐(S727), STAT1 P‐(Y701), STAT3 P‐(S727), and STAT3 P‐(Y705). Total STAT1 and STAT3 protein concentrations were assessed using antibodies recognizing the protein of interest regardless of its phosphorylation state as previously described.^14^ Finally, β‐actin protein concentrations were assessed as a loading control in all samples for normalization of results (Santa Cruz Biotechnology, Inc, Dallas, Texas). All antibodies except β‐actin were purchased from a single source (Cell Signaling Technology, Inc, Danvers, Massachusetts). Briefly, lamellar protein samples (20 μg) were denatured by boiling in SDS/β‐ME buffer (Bio‐Rad; Hercules, California) for 5 minutes, separated by electrophoresis on a polyacrylamide gel (Bio‐Rad) and transferred to a poly(vinylidene fluoride) membrane (Bio‐Rad). The membrane was blocked in 5% milk (Bio‐Rad) in tris‐buffered saline plus Tween‐20 (0.1 vol%/vol% Tween 20; TBST; Thermo Scientific) for 1 hour at room temperature, rocking. The membrane was then incubated with primary antibody against the phosphoprotein of interest at 4°C overnight (STAT1 P‐[S727], STAT1 P‐[Y701] 1 : 1000 dilution; STAT3 P‐[S727] 1: 1200 dilution; and STAT3 P‐[Y705] 1 : 1500 dilution). The membrane was then washed 3 times in 0.1% TBST and incubated with the appropriate IgG horseradish peroxidase‐conjugated secondary antibody for 1 hour at room temperature, rocking (STAT1 P‐[S727], STAT1 P‐[Y701], STAT3 P‐[S727] and STAT3 P‐[Y705] 1 : 10 000 dilution). The membrane was washed in 0.1% TBST, and signal was developed with a chemiluminescent substrate (Thermo Scientific). The membrane was then stripped with a commercially available buffer (Thermo Scientific) and reprobed sequentially for the corresponding total protein and β‐actin (1 : 2000 dilution). Luminescence was measured using a computer software program (US National Institutes of Health, Bethesda, Maryland), and the signal strength was determined by dividing the net intensity of the phosphoprotein (or total protein) band by that of the β‐actin band.

### 2.6 Data analysis

All analyses were conducted using Stata 15.1MP (StataCorp, College Station, Texas) with *P* < .05 as the criterion for statistical significance. Shapiro‐Wilk tests showed that normalized copy numbers for each inflammatory mediator and protein concentration data were not normally distributed. Thus, AMB versus CDH limbs were compared by Wilcoxon signed ranked tests. Data are expressed as median and interquartile range.

## 3 RESULTS

### 3.1 EHC model

All horses developed incessant weight shifting (Obel grade 1 laminitis) 28 to 34 hours after initiating the EHC, at which time each horse was administered phenylbutazone (Nabudone P, Troy Laboratories, Glendenning, Australia) 4 mg/kg bwt IV. Five of the horses received a second dose 8 to 10 hours later. A detailed description of the lamellar histopathology has been published.^3^

### 3.2 Real‐time qPCR

Lamellar mRNA concentrations of CXCL6, CXCL8, IL‐6, IL‐1β, IL‐11, and COX‐2 were decreased in CDH limbs compared to AMB limbs (*P* < .05; Table 1). Lamellar mRNA concentration of COX‐1 was increased in CDH limbs compared to AMB limbs (*P* = .008). Lamellar concentrations of CXCL1, MCP‐1, MCP‐2, TNF‐α, E‐Selectin, and ICAM‐1 mRNA were not significantly different (*P* > .05).

**TABLE 1:** Real‐time quantitative PCR copy number data

Gene	EHC AMB	EHC CDH	Fold change	*P* value
COX‐1	75 200 (52 950‐99 450)	273 000 (99 450‐345 000)	3.63	**.008**
COX‐2	8140 (5565‐18 100)	563.5 (230.8‐811)	.069	**.008**
CXCL1	14 000 (5265‐23 700)	5665 (1875‐11 795)	.40	.15
CXCL6	3425 (1540‐5660)	449.5 (284.5‐1340)	.13	**.02**
CXCL8 (IL‐8)	7750 (2185‐17 350)	352.5 (256.5‐535)	.045	**.008**
E‐selectin	6490 (5418‐9550)	10 565 (5113‐28 800)	1.63	.15
ICAM‐1	4935 (4170‐6633)	5805 (3643‐16 825)	1.18	.15
IL‐1β	15 900 (8295‐31 300)	2860 (1250‐3308)	.18	**.008**
IL‐6	81 800 (27 325‐292 500)	1170 (170‐2240)	.014	**.008**
IL‐11	4070 (1323‐6950)	294.5 (179.5‐687.3)	.072	**.008**
MCP‐1	2520 (1378‐3275)	622.5 (144.8‐1128)	.25	.05
MCP‐2	56 950 (49 100‐83 925)	40 650 (22 425‐77 500)	.71	.46
TNF‐α	37 350 (30 450‐44 050)	59 600 (30 100‐98 675)	1.60	.05

*Note:* Copy number data evaluated with Wilcoxon signed rank tests, presented as median (IQR). Fold change calculated as EHC CDH/median EHC AMB for each gene of interest. Values in bold type are those that are significantly different between EHC AMB and EHC CDH (*P* < .05).

Abbreviations: AMB, ambient temperature; CDH, continuous digital hypothermia; EHC, euglycemic‐hyperinsulinemic clamp; IQR, interquartile range; PCR, polymerase chain reaction.

### 3.3 Western immunoblotting

**FIGURE 1:**
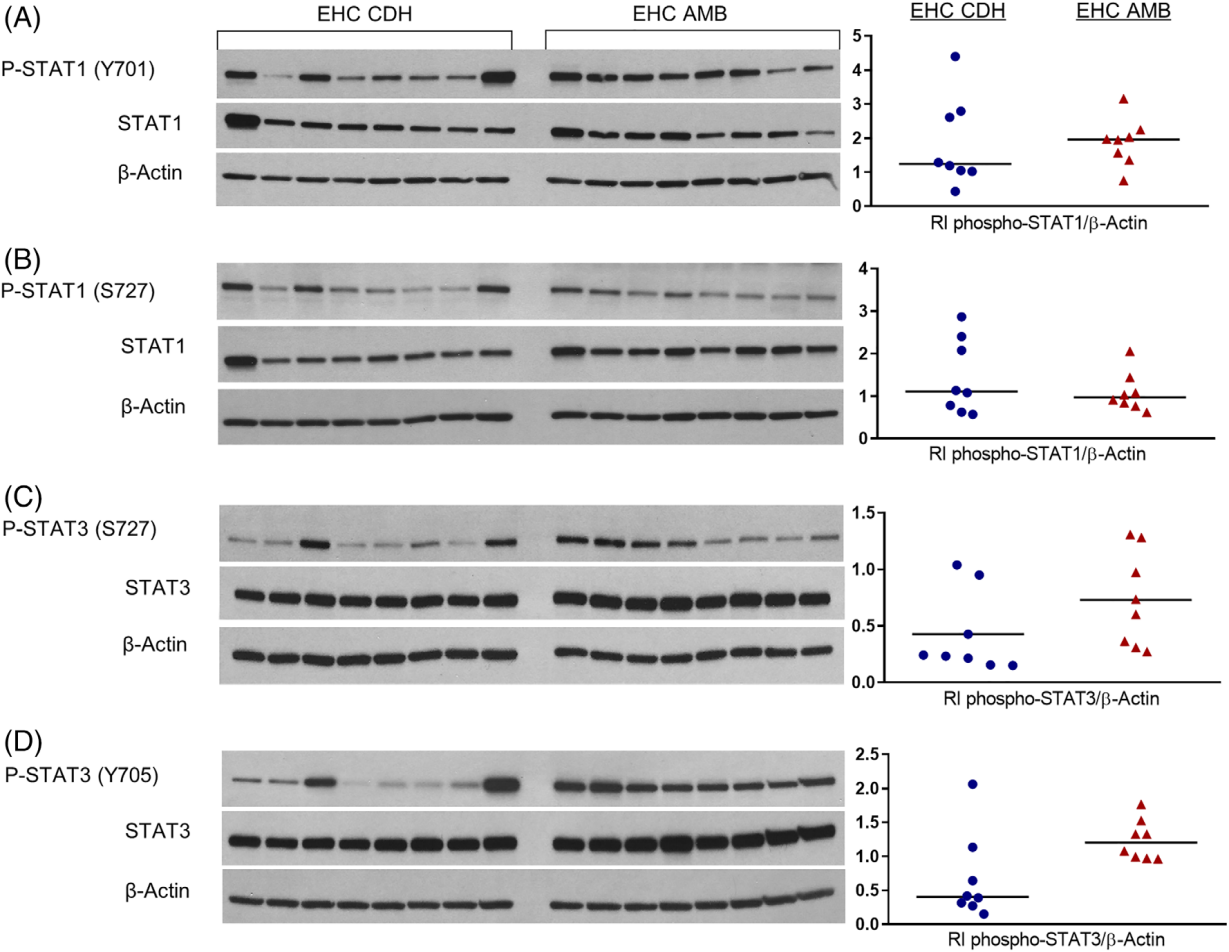
Western immunoblot data evaluating lamellar concentrations of phosphorylated and total STAT1 protein (A, B) and STAT3 protein (C, D) in horses that underwent and EHC with 1 forelimb continuously cooled (CDH) and the other kept at AMB. CDH limbs had a lower concentration of total STAT3 protein compared to AMB limbs (*P <* .001). There was no difference in lamellar concentrations of total STAT1 protein (*P* = .75) or phosphorylated STAT proteins (P‐STAT1 [S727] *P* = .25; P‐STAT1 [Y701] *P* = .64; P‐STAT3 [S727] *P* = .19; P‐STAT3 [Y705] *P* = .05) between CDH and AMB limbs. AMB, ambient temperature; CDH, continuous digital hypothermia; EHC, euglycemic‐hyperinsulinemic clamp; P‐STAT, phosphorylated STAT; RI, relative band intensity; STAT1, signal transducer and activator of transcription 1; STAT3, signal transducer and activator of transcription 3

Lamellar concentration of total STAT3 protein was decreased in CDH limbs compared to AMB limbs (*P* < .001; Table 2). There was no change in lamellar concentrations of total STAT1 protein (*P* = .75) or phosphorylated STAT proteins (P‐STAT1 [S727] *P* = .25; P‐STAT1 [Y701] *P* = .64; P‐STAT3 [S727] *P* = .19; P‐STAT3 [Y705] *P* = .05) in CDH limbs compared to AMB limbs (Figure 1).

**TABLE 2:** Western immunoblot relative intensity data for concentrations of phosphorylated and total signal proteins (STAT1 and STAT3)

	EHC AMB	EHC CDH	*P* value
Lamellar phosphorylated signal protein concentrations			
P‐STAT1 (S727)	0.97 (0.79‐1.35)	1.11 (0.67‐2.32)	.25
P‐STAT1 (Y701)	1.96 (1.41‐2.19)	1.24 (1.03‐2.74)	.64
P‐STAT3 (S727)	0.67 (0.32‐1.20)	0.24 (0.17‐0.82)	.19
P‐STAT3 (Y705)	1.20 (0.98‐1.48)	0.41 (0.28‐1.01)	.05
Lamellar total signal protein concentrations			
STAT1	1.30 (0.96‐1.42)	1.14 (0.95‐1.50)	.75
STAT3	1.62 (1.48‐1.69)	1.17 (1.11‐1.29)	**<.001**

*Note:* Western immunoblot relative intensity data evaluated with Wilcoxon signed rank tests, presented as median (IQR). Statistical significance accepted at *P* < .05. Values in bold type are those that are significantly different between EHC AMB and EHC CDH (*P* < .05).

Abbreviations: AMB, ambient temperature; CDH, continuous digital hypothermia; EHC, euglycemic‐hyperinsulinemic clamp; IQR, interquartile range; STAT1, signal transducer and activator of transcription 1; STAT3, signal transducer and activator of transcription 3.

## 4 DISCUSSION

In a recent study in Standardbred horses that underwent the EHC model of laminitis, lamellar mRNA concentrations of inflammatory mediators, IL‐6, IL‐1β, IL‐11, and COX‐2, were shown to be increased, and this was suggested to be evidence of cross talk between inflammatory and metabolic regulatory signaling pathways.^14^ In our study, CDH inhibited this increase in lamellar mRNA concentrations of inflammatory mediators, indicated by decreased lamellar mRNA concentrations of IL‐6, IL‐1β, IL‐11, and COX‐2 in CDH limbs compared to AMB limbs. This finding is consistent with the results of a study that investigated the effects of CDH on early lamellar inflammatory signaling in sepsis‐related laminitis (oligofructose [OF] model).^16^ In the OF model, CDH decreased lamellar mRNA concentrations of the same inflammatory mediators observed to be decreased in our study.^16^ Importantly, it is thought that the anti‐inflammatory effects of CDH help to reduce the severity of acute lamellar injury in sepsis.^16^ Furthermore, it has been suggested that cell signaling mechanisms upstream of lamellar inflammatory mediators could represent potential pharmacological targets^15^, ^16^ particularly IL‐6/gp130 signaling, which may be activated in the EHC and OF laminitis models.^15^ Indeed, the decreased lamellar mRNA concentration of IL‐6 observed in CDH limbs in our study supports that inhibition of IL‐6/gp130 signaling could be associated with reducing the severity of lamellar injury through inhibition of mTORC1 and STAT3 activation.^9^, ^15^, ^17^

STAT1 and STAT3 play an important role in the development of inflammatory disease by serving as transcription factors, which result in the production of various inflammatory mediators.^19^, ^20^ Evidence of STAT3 activation has been observed in the EHC^14^ and OF laminitis models^15^ and it has been hypothesized that STAT3 is activated via IL‐6/gp130 signaling.^15^ Furthermore, it has been suggested that STAT3 activation is associated with loss of lamellar epithelial cell adhesion molecules (ie, desmosomes) in the EHC model and that inhibition of STAT3 activation via mTORC1 inhibition could protect the epidermal lamellae from structural failure.^15^ Our study found that lamellar concentrations of activated STAT1 and STAT3 were not significantly different between CDH and AMB limbs, suggesting that this might not be a major therapeutic mechanism of CDH in the EHC model. However, total STAT3 concentrations were significantly lower in CDH limbs compared to AMB limbs, which is consistent with downregulation of STAT3 expression in CDH limbs. This finding could be associated with the decrease in IL‐6 lamellar mRNA concentrations observed in our study, as IL‐6 induces STAT3 gene expression.^21^, ^22^

COX‐2 is induced by pro‐inflammatory cytokines, growth factors, and oncogenes, and its overexpression is associated with the inflammatory response and carcinogenesis.^23^, ^24^, ^25^, ^26^, ^27^ Lamellar mRNA concentration of COX‐2 is markedly increased in the EHC and OF laminitis models consistent with COX‐2 overexpression because of local inflammatory response and/or growth factor signaling.^14^, ^16^, ^28^ Furthermore, it has been hypothesized that overexpression of COX‐2 could play an important role in the pathogenesis of laminitis by potentiating epithelial‐to‐mesenchymal transition,^28^ an event that has been linked to the pathogenesis of laminitis in association with activation of both p70S6 kinase and RPS6 downstream of the mTORC1 signaling pathway.^9^, ^15^ While it has been suggested that COX inhibitors may represent a therapeutic target for the treatment and prevention of laminitis,^28^, ^29^ it is unknown whether the inhibition of lamellar COX‐2 alone could prevent the development of insulin‐induced laminitis. In our study, it was found that lamellar mRNA concentrations of COX‐2 were lower in CDH limbs compared to AMB limbs, a finding also observed in the OF model.^17^ IL‐1β is a pro‐inflammatory cytokine that is mainly produced by macrophages and fibroblasts (the predominant cell type in dermal lamellae) and increases the expression of other inflammatory mediators including COX‐2, IL‐6, and matrix metalloproteinases, which are associated with the degradation and remodeling of tissues.^30^ Importantly, lamellar IL‐1β expression has been shown to be increased in the EHC laminitis model.^14^ In our study, lamellar mRNA concentration of IL‐1β was decreased in CDH limbs compared to AMB limbs, consistent with an anti‐inflammatory effect of CDH. Furthermore, this could explain lower lamellar COX‐2 mRNA concentrations in CDH limbs. The early administration of phenylbutazone could have also influenced the lamellar inflammatory response observed; however, our study compared the effect of treatment between limbs (rather than between horses), and therefore it is unlikely that phenylbutazone administration significantly affected the outcome and conclusions.

Although the findings of our study suggest that CDH exerts anti‐inflammatory effects in the EHC model, it remains unclear whether this is a primary therapeutic effect (itself preventing development of the laminitis lesions) or whether other disparate effects of CDH are responsible for ameliorating the lesions and therefore the severity of secondary inflammation. Hypothermia has dramatic lowering effects on enzymatic activity and diffusion rates in mammalian cells,^31^, ^32^ and it has been shown that CDH exerts a hypometabolic effect within lamellar tissue in the EHC model, which may limit the development of laminitis lesions by decreasing the rate of lamellar structural protein turnover.^33^, ^34^ In support of this, hypothermia decreases the rate of protein turnover in mammalian cells,^31^ and in studies of experimental endocrinopathic laminitis,^3^, ^35^ epithelial cell stretch and lengthening of secondary epidermal lamellae are key histologic lesions that are consistent with altered turnover of lamellar structural proteins (ie, cell adhesion molecules, cytoskeletal proteins, and extracellular matrix proteins). Importantly, these histologic lesions are limited by CDH in the EHC model,^3^, ^35^ and they precede basement membrane dysadhesion and leukocyte infiltration,^36^ which highlights both the importance of altered lamellar structural protein turnover in the pathogenesis and its potential as a therapeutic target.

Prior EHC and OF model studies have observed minimal lamellar leukocyte infiltration despite the presence of lamellar pathology,^14^, ^36^, ^37^ with the exception being when severe lamellar pathology has been observed.^3^, ^37^, ^38^ Histologically, lamellae from AMB limbs in our study were characterized by moderate to severe dermo‐epidermal separation, BM dysadhesion, and keratinocyte necrosis; thus, the marked leukocyte infiltration was likely a response to these primary events.^3^ Lamellar mRNA concentrations of chemokines CXCL6 and CXCL8 were decreased in CDH limbs in the our study, which may have limited lamellar leukocyte infiltration. Indeed CDH has many other therapeutic effects on various cellular pathways that are not necessarily independent of inflammatory events; for example, it has been demonstrated that CDH reduces lamellar perfusion and lamellar energy metabolism both in normal horses and horses undergoing the EHC.^33^ Importantly, it has been noted that signaling pathways responsible for nutrient sensing and metabolism, including AMPK and mTOR, intersect with inflammatory pathways and therefore may influence each other in the EHC model.^14^ Furthermore, inflammation has been shown to alter protein homeostasis and increase amino acid metabolic demands,^39^, ^40^ which could further contribute to the disruption of lamellar structural protein homeostasis (dysregulation of the synthesis and degradation of cell adhesion molecules, cytoskeletal proteins and extracellular matrix proteins) that causes stretch, loss of lamellar epithelial cell adhesion, and extracellular matrix degradation in laminitis.^3^, ^35^ Similarly, mechanical influences on tissue can stimulate the local expression of inflammatory mediators (particularly COX‐2), as it has been shown in fibroblasts that cyclical stretch (and also the magnitude of stretching) increases COX‐2 expression.^30^, ^41^ This suggests that unnatural mechanical forces acting on components of the lamellar tissue as a consequence of lamellar failure (stretch and separation) may also influence inflammatory gene expression.

A limitation of our study from a clinical perspective was that CDH was applied at the commencement of the EHC before the development of clinical signs of laminitis, and it remains unclear whether CDH applied during acute laminitis (initiated after the onset of lameness) would have a therapeutic effect. Interestingly, although such a protective effect was observed in the OF model when CDH was applied after lameness developed,^42^ CDH did not inhibit increases in lamellar mRNA concentrations of inflammatory mediators, suggesting that this was not an important therapeutic mechanism during the acute phase of sepsis‐related laminitis.^43^ It is important to recognize, however, that the findings of that study as well as our study are limited by the fact that mRNA concentrations were investigated as opposed to quantification of peptides/proteins of interest in their biologically active forms. Therefore, the functional activity and role of inflammatory events could not be fully elucidated.

In conclusion, our study demonstrated that CDH inhibited lamellar inflammatory signaling in the EHC model of endocrinopathic laminitis. Further studies that investigate the cause, timeline, and role of lamellar inflammatory signaling in the pathogenesis of different forms of laminitis are warranted.

## CONFLICT OF INTEREST

Authors declare no conflict of interest.

## OFF‐LABEL ANTIMICROBIAL DECLARATION

Authors declare no off‐label use of antimicrobials.

## INSTITUTIONAL ANIMAL CARE AND USE COMMITTEE (IACUC) OR OTHER APPROVAL DECLARATION

The project was approved by The University of Queensland Animal Ethics Committee (AEC) that monitors compliance with the Animal Welfare Act (2001) and The Code of Practice for the care and use of animals for scientific purposes (current edition). All animals were monitored continuously by the investigators.

## HUMAN ETHICS APPROVAL DECLARATION

Authors declare human ethics approval was not needed for this study.
